# Deep null fixing on optimal compromise among sum and difference patterns of thinned arrays through the Schelkunoff unit circle representation

**DOI:** 10.1038/s41598-022-16547-y

**Published:** 2022-07-16

**Authors:** Mateo Raíndo-Vázquez, Juan Antonio Rodríguez-González, María Elena López-Martín, Francisco José Ares-Pena

**Affiliations:** 1 Radiating Systems Group, Department of Applied Physics, University of Santiago de Compostela, 15782 Santiago de Compostela, Spain; 2 Department of Morphological Sciences, University of Santiago de Compostela, 15782 Santiago de Compostela, Spain

**Keywords:** Electrical and electronic engineering, Applied physics

## Abstract

Difference far-field patterns represent a way for pin-pointing a target in both azimuth and elevation, extremely useful in radar applications. At the present work, an innovative method for synthesizing good compromise solutions among sum and difference patterns providing low complexity of the antenna feeding network for uniform thinned arrays is addressed. This procedure uses a hybrid version of the Simulated Annealing algorithm (hybrid SA) to optimize a cost function of radiation characteristics for both sum and difference patterns as peak directivity and side lobe level (SLL) while fixing deep nulls. In this framework, examples of half-wavelength spaced linear arrays from 40 to 120 elements were analyzed, as well as an extension to planar arrays by means of separable distributions was developed. The performance of the method is analyzed with different examples and its potential outlined, showing the ability of fixing deep nulls in both sum and difference patterns which share the same uniform excitation relative amplitudes.

## Introduction

Difference patterns are radiation far-field patterns with huge potentials on many radar application techniques, since these, paired with sum patterns, represent a useful solution to be implemented by search-and-track antennas^1^^(pp. 129–130),^^2^^(chapter 2),^^3^. More precisely, the performance of these tracking techniques is based on a first estimation, performed by the sum mode (for instance, the two halves of a linear array excited in phase) for acquisition and a second stage performing an accurate description of the azimuthal and polar positions by means of two spatially orthogonal difference patterns (where the two halves of the same linear array are out of phase). This approach is developed by the denominated monopulse radars, since they use a unique pulse (just changing phases) for detection purposes, in opposition to other conventional techniques as, for instance, the denominated conical scan or sequential lobing ^4^^(pp. 152–159)^. Classical solutions on the synthesis of difference patterns were developed by Bayliss^5^ defining the basis of these problems from a continuous line source point of view. This approach represents an extension of the seminal technique envisaged by Taylor on the design of sum patterns which presents narrow main beams and symmetric low sidelobes^6^. Afterwards, Elliott introduced modifications in this analytical procedure for controlling the sidelobe topography more accurately^7^. In a quite recent approach and concerning compromise solutions between sum and difference patterns, Álvarez-Folgueiras et al. described a methodology for synthesizing low side lobe sum and difference patterns with a common aperture zone^8^. Beyond the continuous problem and concerning state-of-the-art developments in array antennas, studies regarding improvements of such type of radiation patterns were performed using different techniques. In this manner, two approaches based on both convex and linear programming for fixed geometry of linear/planar arrays were addressed by Bucci et al.^9^. On the other hand, the use of sub-arrays has received some attention since it allows to increase the number of degrees of freedom of the problem by introducing a little variability on the excitations of the array and moderately affecting the feeding network complexity. In these works, including the boundary condition for improving both sum and difference patterns produced by the same set of excitations (i.e., addressing the compromise of both sum and difference patterns) within the synthesis problem, solutions by means of sub-arraying techniques can be discussed. As a first example, for the linear case, McNamara discussed the production of the “best compromise” difference pattern from an optimum sum one in terms of side lobe level (SLL)^10^. Improving this strategy, optimization techniques involving a cost function via the minimization of the SLL in both sum and difference modes through Simulated Annealing techniques were purposed by Ares et al.^11,12^. In this same line, López et al. developed a joint optimization of subarray configurations and element excitations^13^, and Caorsi et al. optimized difference patterns of monopulse antennas by considering a hybrid real/integer differential evolution algorithm^14^. On the other hand, Morabito et al. addressed this problem by developing techniques based on convex programming^15^. Alternatively, Mohammed developed a procedure based on Iterative Fast Fourier transform (IFFT) resulting in low complexity of the feeding network by sharing element excitations in both modes^16^. Additionally, a procedure for designing forward-looking monopulse arrays capable of reconfiguring the array from the sum to the difference mode by altering the position of a group of parasitic elements was proposed by Rocca et al.^17^. Other approaches, based on time modulation strategies were developed by Fondevila et al.^18^. Finally, an approach which addressed the possibilities of optimizing sum-difference compromises fixing quasi nulls in their patterns was developed by Rodriguez et al.^19^. In this case, phase only and sub-arraying were considered but it is important to highlight that also here (as well as all the previous cases) no uniform amplitudes were considered for excitations. Regarding array thinning techniques and considering uniform arrays, Haupt proposed interleaved arrays by developing three different approaches (one full and two partially modes of interleaving) by means of genetic algorithms (GA)^20^. Thus, following the spirit of a very recent publication^21^ where improvements on the null fixing for pencil beam patterns by means of array thinning in uniformly excited arrays were proposed, the present paper introduces an innovative technique for developing improvements on compromises between sum and difference far-field patterns.

Previous methods^10–20^ either only study the optimization of some of the described characteristics of the pattern (SLL and directivity) or they do not use array thinning in order to simplify the feeding network. Also, none of the above-mentioned articles can fix deep, analytical nulling directions in the pattern, with the exception of^21^, which does not analyze the compromise between sum and difference patterns. Thus, the presented method overcomes state-of-the-art techniques, since it represents the first approach of addressing the optimization of SLL and directivity of thinned antenna arrays including deep null fixing in the compromise involving sum and difference patterns at the same time. As a consequence, implications on radar applications are addressed. Additionally, studies on the generalization of these linear arrays for the planar case, through the using of the separable distributions paradigm, are discussed.

## Background theory and methods

### Antenna array factor

The expression of a general array factor $F\left(\theta ,\upphi \right)$ considering $2N$ identical oriented radiating elements is^1^

1 $$F\left(\theta ,\varphi \right)=\sum_{n=1}^{2N}{I}_{n}{e}^{jk\left({x}_{n}\mathit{sin}\theta \, \mathit{cos}\varphi +{y}_{n}\mathit{sin}\theta \,  \mathit{cos}\varphi +{z}_{n} \, \mathit{cos}\theta \right)}$$

where $k$ is the wavenumber; ${I}_{n}$ the complex relative excitation of the $n$-th element; $({x}_{n},{y}_{n},{z}_{n})$ the position of the feed point; while $j$ represents the imaginary unit; and $\theta$ and $\varphi$ are the polar and azimuthal angles, respectively.

### Linear arrays

Now, without loss of generality, and for analyzing the cases involved in this work, the origin of the coordinate system will be positioned at the array center. In such a way, particularizing for an equally spaced linear array with the elements at the positions $\left({x}_{n},{y}_{n},{z}_{n}\right)=\left(\mathrm{0,0},\pm nd\right),$ being $d$ the spacing between the elements, the expression of the array factor is simplified to

2 $$F\left(\theta \right)=\sum_{n=-N}^{-1}{I}_{n}{e}^{j\left(n+1/2\right)kd \, \mathit{cos}\theta }+\sum_{n=1}^{N}{I}_{n}{e}^{j\left(n-1/2\right)kd \, \mathit{cos}\theta },$$

producing a $\varphi$-symmetric pattern.

Regarding the nature of the far field pattern produced by the linear array, two particular cases are interesting for developing the array thinning techniques proposed in these studies: cases producing sum and difference far field patterns.

For sum patterns, the complex relative excitation of the radiating elements which generate the far field pattern has to be symmetrical (i.e., ${I}_{-n}={I}_{n}$). In such a manner, the array factor becomes

3 $$F\left(\theta \right)=\sum_{n=1}^{N}{I}_{n}\left[{e}^{j(n-1/2)kd \, \mathit{cos}\theta }+{e}^{-j(n-1/2)kd \, \mathit{co}s\theta }\right],$$

and then, simplifying the expression, the array factor corresponds to

4 $$F\left(\theta \right)=2\sum_{n=1}^{N}{I}_{n} \, \mathit{cos}\left[(n-1/2)kd \, \mathit{cos}\theta \right].$$

On the other hand, in the case of linear arrays generating difference patterns, the complex relative excitation of the elements is anti-symmetrical, thus both halves of the linear array are excited with a symmetrical relative amplitude and in phase opposition (i.e., ${I}_{-n}=-{I}_{n}$ or ${I}_{-n}={I}_{n}{e}^{j\pi }$). Therefore, manipulating the array factor, one can find that

5 $$F\left(\theta \right)=\sum_{n=1}^{N}{I}_{n}\left[{e}^{j(n-1/2)kd \, \mathit{cos}\theta }-{e}^{-j(n-1/2)kd \, \mathit{cos}\theta }\right],$$

and then, simplifying the expression, it can be expressed as

6 $$F\left(\theta \right)=2j\sum_{n=1}^{N}{I}_{n}\mathit{sin}\left[(n-1/2)kd \, \mathit{cos}\theta \right].$$

Therefore, as the symmetry/anti-symmetry of the patterns are assumed, only one half of the arrays are considered for the optimization process.

Finally, it is worth highlighting that, in order to maximize the simplicity of the feeding network, the linear arrays here analyzed will present uniform relative amplitudes. In this manner, the array thinning strategy (elements set to zero or one) will take place.

### Peak directivity of linear arrays

The peak directivity of an array can be used as quality parameter. In such a way, it provides an idea about the performance of a linear array generating a certain far field pattern. The general expression, on linear arrays, can be expressed as (^1^^(pp. 153–154)^)

7 $${D}_{max}=\frac{2F\left({\theta }_{max}\right){F}^{*}({\theta }_{max})}{{\int }_{0}^{\pi }F\left(\theta \right){F}^{*}\left(\theta \right)\mathit{sin}\theta d\theta }$$

where ${\theta }_{max}$ is the maximum radiation angle.

Then, applying the description of sum patterns, the expression of the peak directivity, considering a spacing of $d=\lambda /2$(being $\lambda$ the wavelength) and the previous description of the array factor (Eq. 4), is simplified to

8 $${D}_{max}=\frac{{\left({\sum }_{n=1}^{N}{I}_{n}\right)}^{2}}{{\sum }_{n=1}^{N}{I}_{n}^{2}}.$$

Alternatively, considering the simplified version of the array factor for difference patterns (Eq. 6), and similarly to the work developed by Hansen concerning continuous aperture distributions^3^, the peak directivity of a difference far-field pattern produced by an out of phase linear array with a spacing of $\lambda /2$, can be expressed as

9 $${D}_{max}=\frac{{\left\{{\sum }_{n=1}^{N}{I}_{n}\mathit{sin}\left[\left(n-\frac{1}{2}\right)\pi  \, \mathit{cos}{\theta }_{max}\right]\right\}}^{2}}{{\sum }_{n=1}^{N}{I}_{n}^{2}}$$

where ${\theta }_{max},$ in this case, is the angular position of one of the main beams of the difference pattern.

In light of the present expressions derived from the general formulation of the peak directivity of an antenna, it is worth noting that the computation time of an optimization process will be drastically reduced by implementing these two simplified equations, valid for linear arrays of half-wavelength spacing, instead of the above-mentioned integral of the far-field pattern.

Then, one can define the normalized peak directivity $(\eta )$ in order to compare different solutions. This parameter $\eta$ is determined by dividing the peak directivity of the pattern by the peak directivity of the uniform case (pattern with all the relative excitations set to 1).

### Optimization procedure

The optimization procedure proposed in the present work implements a hybrid SA algorithm^23^, which combines the local optimization method of the downhill simplex with a slowly reducing temperature parameter from the Simulated Annealing algorithm, resulting in a global optimization method. The proposed procedure codifies the relative amplitudes of the elements of the antenna as a sequence of zeros and ones (i.e., modelling an array thinning strategy). This encoding is performed by normalizing every continuous value of the relative amplitudes, ranging from zero to one, and setting to bit-zero the values lower than 0.5 and to bit-one those equal or greater than 0.5. GA-based alternatives are excluded, since they present greater computational cost associated (something particularly dramatic for a high number of array elements), as demonstrated already in^21^.

The sequence of codified relative amplitudes is iteratively modified by the hybrid SA algorithm in order to find the combination whose characteristics match the desired ones in both the sum pattern (symmetric phase) and the difference pattern (anti-symmetric phase). More precisely, the procedure follows a similar strategy as the already described in^21^, but generalizing its use to compromise solutions among sum and difference far-field patterns. In such terms, a cost function is defined, where each chosen parameter of both the sum and difference radiation patterns are implemented. The value of this cost function grows with the deviations of the characteristics of the patterns from the desired values. Thus, the algorithm is set to reduce the value of such cost function in order to optimize both the SLL and the peak directivity of the far field pattern on both sum and difference modes, while fixing some number of deep nulls by means of the Schelkunoff unit circle representation of the array factor^22^.

To this aim, a general cost function for the process can be defined as

10 $$C={C}_{sum}+{C}_{difference}$$

where ${C}_{sum}$ and ${C}_{\mathrm{difference}}$ are determined by particularizing a ${C}_{pattern}$ for each case, being

11 $${C}_{pattern}={c}_{1}{\left|SL{L}_{o}-SL{L}_{d}\right|}^{2}H\left(SL{L}_{o}-SL{L}_{d}\right)+{c}_{2}{\left|{\eta }_{o}-{\eta }_{d}\right|}^{2}H\left({\eta }_{o}-{\eta }_{d}\right)+{c}_{3}{\sum }_{i=1}^{M}\left|{\theta }_{0,i}^{o}-{\theta }_{0,i}^{d}\right|$$

where $SL{L}_{o}$ and $SL{L}_{d}$ are the obtained and desired SLL, respectively; ${\eta }_{o}$ and ${\eta }_{d}$ are obtained and desired normalized peak directivity on ${\theta }_{max}$; $H(\cdot )$ is the Heaviside step function (^24^^(p. 1020)^); $M$ corresponds to the number of desired nulls to be fixed; ${\theta }_{0,i}^{o}$ and ${\theta }_{0,i}^{d}$ are the obtained and desired null position by means of their polar angles and, finally, ${c}_{1},{c}_{2},$ and ${c}_{3}$ are the different weights of the cost function. The polar angles of these null positions were obtained by introducing the Schelkunoff unit circle representation of the roots (${\omega }_{n}$) of the polynomial associated to the relative excitations, as pointed out in^21^. In such a way, the angular position of each null of the far field pattern ${\theta }_{0,n}$ was calculated after determining the ${\psi }_{0}^{o}$ angle of ${\omega }_{n}$ (represented in the complex plane), since ${\theta }_{0,i}^{o}=acos({\psi }_{0}^{o}/kd).$

### Extension to planar arrays

For the case of the extension to planar architectures, the approach here developed is based on the principle of separable distributions^25^. The relative excitations for the planar array are calculated by first laying our already optimized linear arrays in both the $x$ and $y$ axes, and then calculating the excitation of any element as the product of the relative excitations of the elements corresponding to the projections in both axes. In such a way, for each one of the two main axes of the 3D far field pattern, a certain SLL, peak directivity and deep null positioning are obtained. So, the array factor considering a separable planar array synthesized from two linear symmetrically excited arrays lying on the $x-y$ plane is given by the multiplication of the array factors of each one of the corresponding linear arrays. For instance, if the simplifications derived previously in (4) for the case of sum patterns are considered, the expression becomes

12 $$F\left(\theta ,\varphi \right)=4\left[\sum_{{n}_{x}=1}^{2{N}_{x}}{I}_{{n}_{x}} \, \mathit{cos}\left[\left({n}_{x}-1/2\right)k{d}_{x} \, \mathit{sin}\theta  \, \mathit{cos}\varphi \right]\right]\cdot \left[\sum_{{n}_{y}=1}^{2{N}_{y}}{I}_{{n}_{y}}\mathit{cos}\left[\left({n}_{y}-1/2\right)k{d}_{y}\mathit{sin}\theta  \, \mathit{cos}\varphi \right]\right].$$

where ${I}_{{n}_{x}}$ and ${I}_{{n}_{y}}$ are the relative excitations of the $x$ and the $y$ axes, respectively.

In this case, the pattern is composed by two sum patterns (^1^^(p. 207)^), since symmetrical normalized current distributions are assumed for each axis.

Alternatively, including a difference pattern in one of the two axes, the tridimensional solutions obtained differ in consequence. More precisely, the expressions of patterns generated by planar arrays with an anti-symmetrical element relative excitation distribution in one of the two main axes are obtained by homologous manipulations and can be simplified as (^1^^(p. 207)^)

13 $$F\left(\theta ,\varphi \right)=4j\left[\sum_{{n}_{x}=1}^{2{N}_{x}}{I}_{{n}_{x}}\mathit{si}n\left[\left({n}_{x}-1/2\right)k{d}_{x}\mathit{sin}\theta \,  \mathit{cos}\varphi \right]\right]\cdot \left[\sum_{{n}_{y}=1}^{2{N}_{y}}{I}_{{n}_{y}} \, \mathit{cos}\left[\left({n}_{y}-1/2\right)k{d}_{y} \, \mathit{sin}\theta  \, \mathit{cos}\varphi \right]\right]$$

and

14 $$F\left(\theta ,\varphi \right)=4j\left[\sum_{{n}_{x}=1}^{2{N}_{x}}{I}_{{n}_{x}} \, \mathit{cos}\left[\left({n}_{x}-1/2\right)k{d}_{x} \, \mathit{sin}\theta  \,  \mathit{cos}\varphi \right]\right]\cdot \left[\sum_{{n}_{y}=1}^{2{N}_{y}}{I}_{{n}_{y}}\mathit{sin}\left[\left({n}_{y}-1/2\right)k{d}_{y}\mathit{sin}\theta \,  \mathit{cos}\varphi \right]\right]$$

respectively. In radar applications, the sum pattern generated in Eq. (12) becomes interesting for acquisition of the target, while both Eqs. (13) and (14) difference patterns are used to boresight the element under tracking more accurately.

Otherwise, in the case of both difference patterns present in both main axes, a double-difference beam, as introduced by Chesley^26^, is addressed.

15 $$F\left(\theta ,\varphi \right)=-4\left[\sum_{{n}_{x}=1}^{2{N}_{x}}{I}_{{n}_{x}}\mathit{sin}\left[\left({n}_{x}-1/2\right)k{d}_{x}\mathit{sin}\theta \mathit{cos}\varphi \right]\right]\cdot \left[\sum_{{n}_{y}=1}^{2{N}_{y}}{I}_{{n}_{y}}\mathit{sin}\left[\left({n}_{y}-1/2\right)k{d}_{y}\mathit{sin}\theta \mathit{cos}\varphi \right]\right]$$

This four-lobed far-field pattern is interesting when dealing with electronic countermeasures^27^ for main beam jamming and accurately estimating the angle of arrival of target^28^.

In the results section, a discussion about the four different 3D patterns obtained by the combination of symmetric/antisymmetric–symmetric/antisymmetric relative excitations distributions of the different linear arrays present on both main axes are addressed.

The peak directivity of the planar arrays, is calculated by following (^1^^(p. 205)^)

16 $${D}_{max}=\frac{4\pi F\left({\theta }_{0},{\varphi }_{0}\right){F}^{*}({\theta }_{0},{\varphi }_{0})}{{\int }_{0}^{\pi /2}{\int }_{0}^{2\pi }F\left(\theta ,\varphi \right){F}^{*}(\theta ,\varphi )\mathit{sin}\theta d\theta d\varphi }$$

where, in this case, the angular position of maximum radiation $({\theta }_{0},{\varphi }_{0})$ of the examples involving difference patterns is the position of the maximum of one of the two main beams (that will be out of broadside direction). As at this stage just an analysis of the obtained results must be performed, there are no necessities for simplifying the expression regarding computation time.

## Results

In the present section, an analysis of the compromise for linear arrays which generate both sum and difference patterns is addressed. Compromise solutions are interesting for radar applications considering feeding network simplifications^11,12^, even if they are not optimum if the problem is treated independently. Thus, in order to analyze the performance of this procedure, a discussion considering radiation characteristics only, with no restrictions of fixing deep nulls, is developed and, afterwards, an analysis of this procedure including null fixing techniques is described. All the examples here reported address equally spaced arrays with $d=\lambda /2.$

### Without null fixing strategies

In order to understand the potentials of the present methodology for obtaining thinned arrays, a range of different sizes were tried. More precisely, linear arrays between 70 and 120 elements were introduced in an optimization process obtaining common solutions for both sum and difference patterns (see Table 1). Cases with less than 70 elements (60, 50, and 40) did not improve the solution with all the elements on.

**Table 1:** Results of the optimization of linear arrays with common relative excitations for generating both sum and difference patterns, improving SLL and peak directivity (${D}_{max}$), without applying deep-null fixing strategies.

# Elements	Far-field pattern type	$SLL$ (dB)	${D}_{max}$ (dB)
70	Sum	− 14.80	18.20
	Difference	− 12.42	15.25
80	Sum	− 15.40	18.57
	Difference	− 14.14	15.57
90	Sum	− 17.45	18.81
	Difference	− 14.00	15.64
100	Sum	− 19.13	19.03
	Difference	− 14.83	15.75
110	Sum	− 18.99	19.14
	Difference	− 14.90	15.81
120	Sum	− 18.95	19.73
	Difference	− 14.23	16.46

On the other hand, it is worth highlighting that the performance of the difference patterns obtained in the compromises is always worst that the complementary sum patterns of the same solution. This happens due to the appearance of the characteristic two main lobes at both side of the null present at $\theta =0$ degrees. In this manner, the value of the peak directivity is reduced to the half of the one of the sum pattern case in natural units. As a result, and as the data reported in Table 1 confirm, a reduction of 3 dB is expected.

### With null fixing strategies

**Figure 1:**
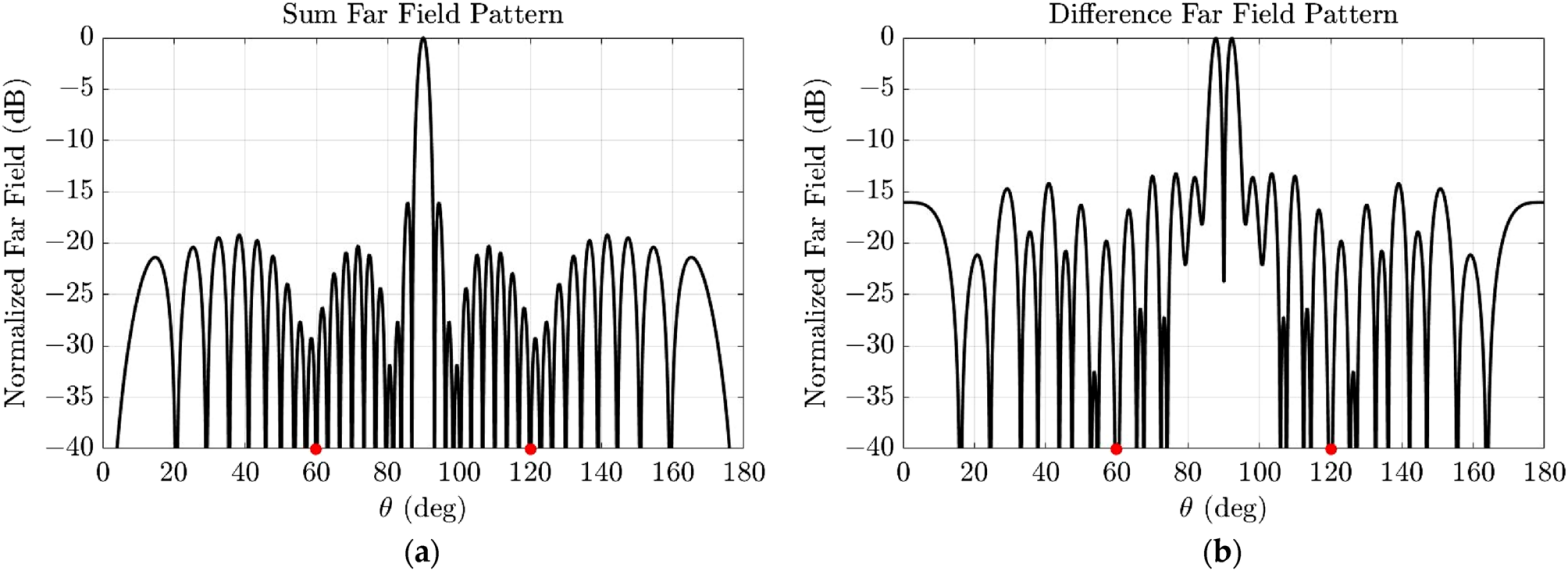
Normalized far field patterns resulting from the compromise solution reported in Table 2 fixing a deep null at 60° and generated by the relative excitations shown in Table 3. (**a**) Sum pattern solution: two halves of the linear array in phase. (**b**) Difference solution: two halves of the linear array out of phase. The red dots indicate the obtained deep null positions.

Considering deep null fixing, 40-element linear arrays were analyzed. In this case, optimizations with desired deep null positions at angles between 40 and 60 degrees, in steps of 5 degrees, were set (see Table 2). From the data obtained, it can be highlighted that solutions with a better null fixing performance show more degradation on the SLL and/or peak directivity of the pattern produced by this linear array. As an example, the case of fixing a common null at 60 degrees is reported in Table 3 and the far-field patterns generated are shown in Fig. 1. Also, in this case, it exists a different of 3 dB approximately between the results of sum and difference patterns obtained for each null fixing, something which is in accordance to the appearance of two main lobes in the case of the difference patterns, while the sum pattern only presents one, as previously discussed.

**Table 2:** Results of the optimization of a linear array of 40 elements with common relative excitations for generating both sum and difference patterns, improving SLL, peak directivity (${D}_{max}$) and including a desired common deep null.

Desired deep null position (deg.)	Far-field pattern type	$SLL$ (dB)	${D}_{max}$ (dB)	Nulls (deg.)
40	Sum	− 14.60	15.80	39.9
	Difference	− 12.08	12.87	40.7
45	Sum	− 14.11	15.56	45.6
	Difference	− 10.66	12.63	44.7
50	Sum	− 9.99	15.31	50.4
	Difference	− 13.54	13.09	50.4
55	Sum	− 13.74	15.80	54.62
	Difference	− 11.47	12.95	55.54
60	Sum	− 16.06	15.56	60.0
	Difference	− 13.21	12.51	60.0

**Table 3:** Right side of the relative excitations vector for the linear arrays for generating a compromise solution for sum (two halves in phase) and difference (two halves out of phase) patterns respectively, fixing a deep null at 60° degrees. The results associated to this solution are reported in Table 2.

Right side of the linear array for the best compromise sum-difference fixing a deep null at 60 degrees	
1 1 1 1 1 1 1 1 1 1 1 1 1 1 1 0 1 0 1 1

### Planar arrays with separable distribution

**Figure 2:**
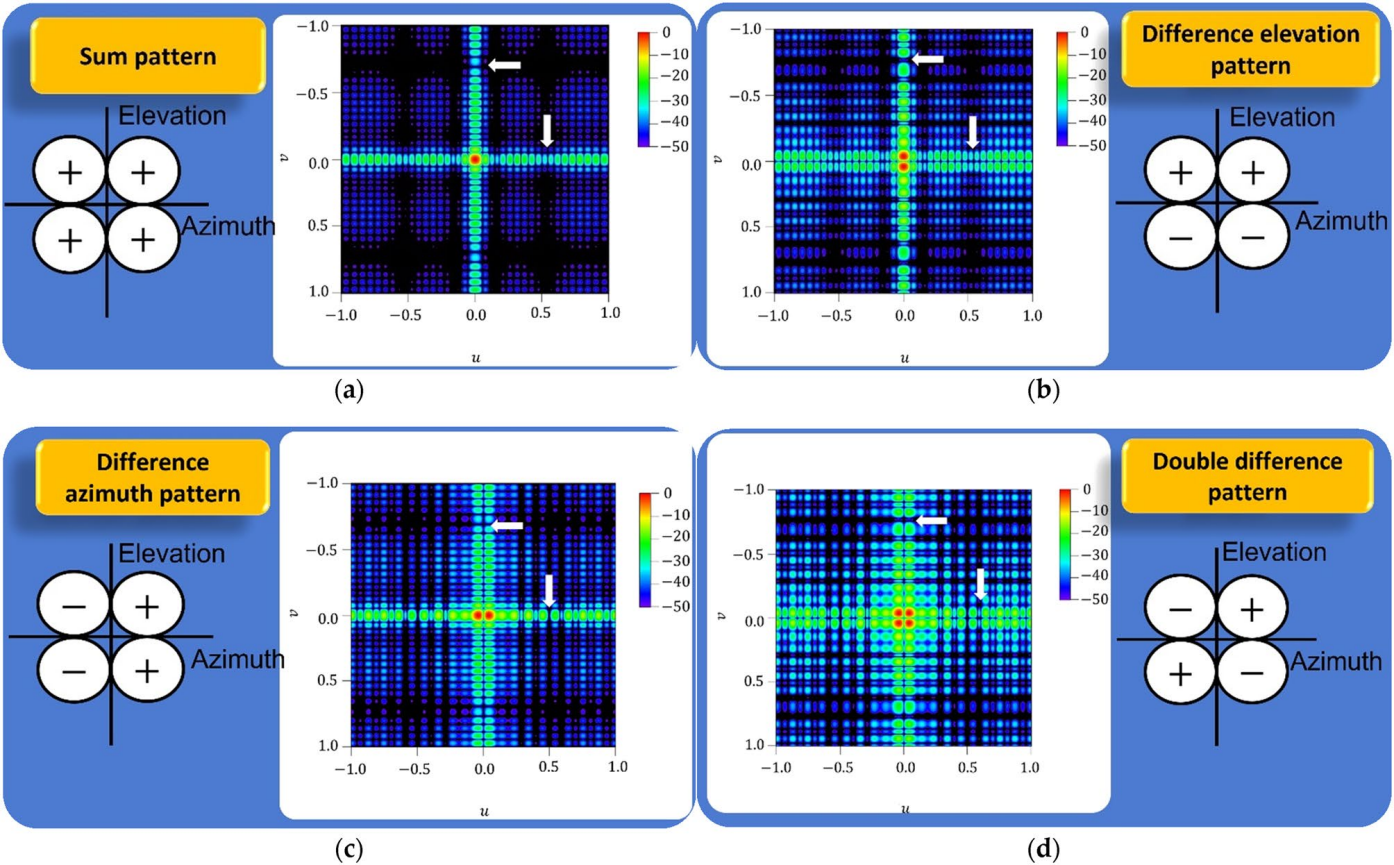
Results for the extension to a planar architecture based on separable distributions. Considering sum/difference pattern on each axis, four results can be produced: (**a**) sum pattern (sum–sum combination), (**b**) elevation difference pattern (difference–sum combination), **(c)** Azimuth difference pattern (sum–difference combination), **(d)** double difference pattern (difference–difference combination). In all the cases, the 3-D representation of the far-field pattern uses is expressed in terms of $u=\mathrm{sin}\theta \mathrm{cos}\varphi$ and $v=\mathrm{sin}\theta \mathrm{sin}\varphi .$ The white arrows included on each tridimensional pattern indicate the obtained deep nulls on each case. The relative phase response in each quadrant is included on each case.

Following the procedure described in the background and methods sections, an extension to planar arrays was developed by means of separable distributions. More precisely, two different cases of null fixing on compromise sum-difference patterns produced by equally spaced (${d}_{x}={d}_{y}=\lambda /2$) linear arrays of 40 elements were used as input of the procedure (i.e., ${N}_{x}={N}_{y}=40$): one of them fixing a deep null at 40 degrees, while the other one fixing it at 60 degrees. These cases were extracted from the results of the previous section, analyzing null fixing strategies in compromise studies (Table 3) and the relative excitations of each linear array are shown in Table 4. In such a way, four cases can be analyzed obtained from the combination of symmetric-antisymmetric linear arrays: sum-sum patterns (Fig. 2a), difference-sum patterns (Fig. 2b), sum-difference patterns (Fig. 2c), difference-difference patterns (Fig. 2d). In the case of both sum patterns a directivity of 35.89 dB was obtained. Alternatively, in the case of difference-sum/sum-difference patterns, the peak directivity was 32.67 dB. Finally, the double difference pattern (the combination of two difference patterns) reached a peak directivity of 29.58 dB.

**Table 4:** Relative excitations vector for the linear arrays used in the design of the planar separable distribution reported in Fig. 2.

Linear array in $\varphi ={0}^{\circ }$	Linear array in $\varphi ={90}^{\circ }$
− 1 − 1 − 1 0 − 1 − 1 − 1 − 1 − 1 − 1 − 1 − 1 − 1 − 1 − 1 − 1 − 1 − 1 − 1 − 1 1 1 1 1 1 1 1 1 1 1 1 1 1 1 1 1 0 1 1 1	− 1 − 1 0 − 1 0 − 1 − 1 − 1 − 1 − 1 − 1 − 1 − 1 − 1 − 1 − 1 − 1 − 1 − 1 − 1 1 1 1 1 1 1 1 1 1 1 1 1 1 1 1 0 1 0 1 1

## Discussion

In the present work, an innovative method for fixing deep nulls in compromises of radiation patterns of linear arrays with symmetrical and anti-symmetrical relative current excitations based on the hybrid SA global optimization algorithm was implemented. More precisely, a procedure already developed for sum patterns was generalized for dealing with finding the best compromise between sum and difference patterns. In such terms, this approach synthesizes a required radiation pattern facilitating the practical realization of the arrays considering their feeding networks. Thus, array thinning and deep null fixing techniques were developed at the same time in the array pattern synthesis for compromises between difference and sum far-field patterns. Regarding computational costs, as it can be expected (similarly to the case addressing sum patterns^21^), the running time differences between the present methodology and GA-based alternatives increase considerably with the number of the array elements.

In order to extend this methodology to a planar architecture, separable distributions were used. As a consequence, different types of 3-D far-field patterns were characterized, considering different combinations between compromise sum/difference patterns generated from each linear array present on each axis. All of them presented fixed nulls in the pattern cuts $\varphi ={0}^{\circ }$ and $\varphi ={90}^{\circ }$ planes. As it is well-know, this methodology presents some limitations since it extends linear array to rectangular grids and boundaries. In light of these considerations, an alternative planar extension based on the collapsed distributions paradigm^25,29^ can be proposed. In such a way, after the generation of some optimized linear arrays guaranteeing some desired characteristics at different cuts of the 3D far-field pattern, the principle of collapsed distributions can be implemented in order to obtain the required relative excitations (± 1 or 0). These are able to be projected on each cut to generate the equivalent linear arrays previously optimized.

## Data Availability

The datasets generated during and/or analyzed during the current study are available from the corresponding author on reasonable request.
